# The Antioxidant Profiles, Lysosomal and Membrane Enzymes Activity in Patients with Acute Pancreatitis

**DOI:** 10.1155/2014/376518

**Published:** 2014-09-15

**Authors:** Halina Milnerowicz, Radosław Bukowski, Monika Jabłonowska, Milena Ściskalska, Stanisław Milnerowicz

**Affiliations:** ^1^Department of Biomedical and Environmental Analysis, Medical University of Wroclaw, Borowska 211, 50-556 Wroclaw, Poland; ^2^Department and Clinic of Gastrointestinal and General Surgery, Medical University of Wroclaw, M. Curie-Skłodowskiej 66, 50-369 Wroclaw, Poland

## Abstract

Oxidative stress and inflammatory mediators, such as IL-6, play an important role in the pathophysiology of acute pancreatitis. The study was aimed to assess the degree of the pro/antioxidative imbalance and estimate which antioxidant plays a role in the maintenance of pro/antioxidative balance during acute pancreatitis. The study was investigated in the blood of 32 patients with acute pancreatitis and 37 healthy subjects. IL-6 concentration as early marker of inflammation was determinated. The intensity of oxidative stress was assessed by TBARS concentration. To investigate antioxidative status, the GPx and Cu/Zn SOD activities and the levels of GSH, MT, SH groups, and TRAP were measured. The concentrations of Cu and Zn as ions participating in the maintenance of antioxidant enzymes stability and playing a role in the course of disease were determinated. The activities of GGT, AAP, NAG, and *β*-GD as markers of tissue damage were also measured. An increase in IL-6 concentration, which correlated with Ranson criteria, and an increase in GPx activity, levels of MT, TBARS, or GGT, and NAG activities in patients group compared to healthy subjects were demonstrated. A decrease in GSH level in patients group compared to control group was noted. The studies suggest that GPx/GSH and MT play the role of the first line of defence against oxidative stress and pro/antioxidant imbalance in the course of acute pancreatitis.

## 1. Introduction

Inflammatory mediators play a key role in acute pancreatitis development and in the systemic complications of the disease, which are a main cause of patient's death [1]. One of the most important mediators of inflammation is interleukin-6 (IL-6), which can be considered as a factor modulating the defence mechanism in organism. It was demonstrated that the IL-6 level is increased in the blood of patients with acute pancreatitis (AP). The concentration of this cytokine was correlated with both the severity of AP and other indicators of inflammation, C-reactive protein and the activity of phospholipase A_2_. The level of IL-6 is an early marker of pancreatitis (24–36 hours after the occurrence of symptoms) and it can be considered as a predictor of disease [1].

Recent results have confirmed an essential role of oxidative stress in pathogenesis and pathophysiology of pancreatitis [2–5]. Numerous studies have suggested an essential role of antioxidants in the course of inflammatory process, among which a significant role plays: glutathione (GSH), glutathione peroxidase (GPx) (EC 1.11.1.9), and Cu/Zn superoxide dismutase (Cu/Zn SOD) (EC 1.15.1.1) [3–7].

Glutathione (glutamyl-cysteinyl-glycine) is mainly known for its important role as a major contributor to the intracellular reducing environment. Along with GPx it participates in the depletion of hydrogen peroxide and other organic peroxides, protecting the cells before protein SH groups, nucleic acids, and lipids oxidation. The role of Cu/Zn SOD in the protection against the GSH depletion, the lipids peroxidation, and the progression of AP was shown [7–9].

The relation between Cu/Zn SOD and GPx in acute pancreatitis is controversial. In experimentally induced acute pancreatitis, a substantial decrease of Cu/Zn SOD activity was detected [8], while GPx activity considerably increased in blood hemolysates and pancreatitis cells [5]. No substantial differences in GPx activity between the control group and the group of patients with AP of were noticed by Szuster-Ciesielska et al. [10]. Cu/Zn SOD activity significantly increased in the serum of patients with AP and chronic pancreatitis [10]. The Cu/Zn SOD activity can influence the level of zinc and copper ions participating in the maintenance of the proper stability of the subunits of enzyme and neutralization of superoxide radical anion. Pancreas plays an important role in the homeostasis of metals, what is confirmed by the changes in the Cu/Zn ratio in the course of the disease [6, 11].

SH groups of proteins play an important role in the maintenance of pro/antioxidative balance. One of them is metallothionein (MT), a cysteine rich (20–33%) small molecular weight protein (6-7 kDa). MT can neutralize reactive oxygen species (ROS) and scavenge free radicals, thereby acting as antioxidant. The role of this protein in maintaining the homeostasis of Zn and Cu was also shown [12].

The ability to scavenge free radicals in organism can be assessed by total peroxyl radical trapping potential (TRAP) determination, which is the combined capacity of all antioxidants to neutralize free radicals in serum. It is a marker to signal the beginning of oxidant-antioxidant imbalance, which results in oxidative stress [13]. Oxidative damage induces cascade of reactive oxygen species production, some of which are relatively transients, such as hydroxynonenol, while others appear later and accumulate, such as malondialdehyde [14].

As a result of intensified pancreatitis and oxidative stress, the tissue damage of pancreas and the increased release of cellular enzymes to extracellular space are observed. The membrane enzymes there are alanine aminopeptidase (AAP) (EC 3.4.11.2) and *γ*-glutamyltransferase (GGT) (EC 2.3.2.2). AAP is a glycoprotein with low content of cysteine residue and high content of Zn ions [15]. It is known as a sensitive indicator of pancreatic disease. GGT is a microsomal enzyme, which catalyzes hydrolysis of the bond linking the glutamate and cysteine residues of glutathione and glutathione-S-conjugates [16]. It was suggested that GGT activity can be considered as a good marker, which allows differing alcohol-related AP from others AP [17]. In other studies, it was shown that alcohol abuse did not cause the increase in GGT activity [18]. N-acetyl-*β*-D-glucosaminidase (NAG) (EC 3.2.1.30) and *β*-glucuronidase (*β*-GD) (EC 3.2.1.31) are known as lysosomal enzymes. A statistically significant increase in NAG activity in pancreatocytes was shown as a result of tissue damage in rats with AP [19]. The determination of *β*-GD activity is a sensitive marker of increased phagocytic activity. The increased *β*-GD activity can suggest coming cell lysis [20].

The aim of the study was to assess the degree of disturbances in the pro/antioxidative balance of above mentioned markers and estimate which antioxidant plays the main role in the maintenance of pro/antioxidative balance during acute pancreatitis. The study was aimed to determine an early marker of pancreatic damage in patients with acute pancreatitis.

## 2. Materials and Methods

### 2.1. Materials

The tests were conducted in the blood, serum, and plasma derived from the patients of Department and Clinic of Gastrointestinal and General Surgery, Medical University of Wroclaw. All hospitalized patients and healthy volunteers of the control group had been informed about the aim of the study and gave their consent. The study protocol was approved by Local Bioethics Committee of Wroclaw University of Medicine (No: KB-257/2005 and KB-829/2012).

The venous blood was collected on the patients' admission to hospital and during their hospitalization. The blood samples were obtained from 32 patients. They were classified into the group of patients with AP due to clinical symptoms, personal interview, physical examination and clinical method used in the diagnosis of pancreatitis (ultrasonography, radiological examinations, and computed tomography of abdominal cavity), and laboratory tests (C-reactive protein, leukocytosis, the activity of amylase, lipase, aspartate and alanine transaminase, alkaline phosphatase, the level of bilirubin, urea, creatinine, and albumin in serum). From the study, were excluded the patients with pancreatic cancer arising in the course of pancreatitis. As control group, 37 healthy volunteers were qualified by clinician of primary medical care. The patients were in age of 53.1 ± 10.6 (4 patients in age of 31–40, 12 in age of 41–50, 6 in age of 51–60, 8 in age of 61–70, and 2 in age of 71–80). The control group was in age of 41.3 ± 10.0.

The serum was collected according to the routine procedure, by taking the venous blood to disposable test tube. The plasma was obtained through collecting blood to a test tube with EDTA or heparin and they were mixed immediately and centrifuged (2500 g/15 min). The serum and plasma were frozen rapidly and stored at −30°C. From the fresh collected blood, samples hemolysates were prepared (150 *μ*L of blood and 1050 *μ*L H_2 _O_mQ_), and then 300 *μ*L 25% metaphosphoric acid (Cat. no: 23 927-5, Sigma-Aldrich, Germany) was added, so that the final concentration of it in hemolysate was 5%. The samples were thoroughly mixed and then centrifuged for 6 minutes (10000 g). The supernatant obtained in this way for GSH analyzing was collected and used.

In the case of some patients with AP, the dynamics of selected examined parameters was performed. Additionally, on the basis of personal interview and medical diagnosis, the group of patients was divided according to the etiology of disease to check the course of disease changes. Two groups of patients (patients with gallstone and idiopathic AP and patients with alcohol-related AP) were distinguished.

### 2.2. Methods

The concentration of IL-6 in serum was determined using DuoSet-ELISA development system test (Cat. no. DY206, R&D Systems, USA) using mice antibodies against human IL-6, immobilized on 96-wells polystyrene plate (Nunc-Immuno Modules MaxiStrip, Cat. no. 468667, Nunc, Germany).

GSH level was determined in blood hemolysates with the method early described using glutathione standard (Cat. no: 49 750 Sigma-Aldrich, Germany) and alloxan (Cat. no: 23 437-0, Sigma-Aldrich, Germany) developed by Patterson and Lazarow [21].

Thiol groups (SH groups) concentration was measured in plasma using a colorimetric method based on the reaction with 2,2-dithiobisnitrobenzoic acid (Ellman's reagent) (Sigma Aldrich, Germany) according the method described earlier [22].

MT concentration in plasma was measured using two-step direct ELISA method with commercial antibody (Dako, Denmark) according the method described by Milnerowicz and Bizoń [23].

Measurements of metals (Cu and Zn) concentrations in the serum were assessed with the use of FAAS method (Flame Atomic Absorption Spectrometry) in the acetylate flame on the SOLAAR M6 apparatus (Thermo Elemental, Solaar House, Cambridge, UK). The accuracy and repetition of the method were verified by determining the metal concentrations in control serum samples (Seronorm TM Trace Elements Serum of Sero AS, Bilingstad, Norway, Cat. no: 201405). On the base of the concentration of Cu and Zn in serum, the Cu/Zn ratio was calculated.

GPx activity in the blood plasma was determined using Glutathione Peroxidase Colorimetric/Kinetic Assay test (Cat. no: CM89031, Immuno-Biological Laboratories, Germany). Serum Cu/Zn SOD activity was determined with Superoxide Dismutase Assay (Cat. no: CM706002, Immuno-Biological Laboratories, Germany).

Total peroxyl radical trapping potential (TRAP) was determined in plasma according to the modified method of Alho and Leinonen [24] and described earlier [25].

The concentration of lipid peroxidation products (thiobarbituric acid reactive substances, TBARS) in the plasma was measured using thiobarbituric acid (TBA; Cat. no: 011.350-6, Sigma-Aldrich, Germany) according to the method described earlier [26].

Serum NAG activity was performed by colorimetric methods with use of p-nitrophenyl-N-acetyl-*β*-D-glucosamide (Cat. no. 222-398-7, Sigma-Aldrich, Germany) as a substrate according to the method of Maruhn [27]. *β*-GD activity in serum was measured according to the method developed by Maruhn et al. [28]. As a substrate, p-nitrophenyl-*β*-D-glucuronide (Cat. no. 233-753-0, Sigma Aldrich, Germany) was used. Serum AAP activity was performed in colorimetric methods using L-alanyl-*β*-naphthylamide (Cat. no. 211-956-5, Sigma-Aldrich, Germany) as substrate according to the method described earlier [29]. GGT activity in serum was measured using Bio-LA-TEST (Cat. no. 1105902, LACHEMIA, Czech Republic) and *γ*-glutamyl-p-nitroanilide as substrates.

### 2.3. Statistical Analysis

Statistical analysis was carried out using the program Statistica 9.0. The results for control group and the group of patients with alcohol-related AP were analysed using Student's* t* test. Normality of distribution was confirmed by Shapiro-Wilk test. In the absence of normal distribution, the nonparametric* U* Mann-Whitney test was used. In order to verify the correlation between parameters, the Spearman correlation was performed. Statistical significance was accepted for *P* < 0.05.

## 3. Results

Results of determination of antioxidant balance parameters were calculated for the first day of hospitalization (Tables 1, 2, and 3).

### 3.1. Serum IL-6 Concentration and Ranson Criteria

More than 55-fold increase in IL-6 concentration in the group of AP was demonstrated compared to control group (Table 1). In AP patients, IL-6 concentration has been increased rapidly within several tens of hours since the occurrence of acute pancreatitis symptoms. The maximum of IL-6 concentration was reached after 2-3 days and thereafter gradually decreased to about 10 U/mL. The Ranson criteria for patients with AP were also assessed (Table 1).

### 3.2. GSH Level in Blood Hemolysates

The results have shown significant decrease in GSH level in blood of the examined patients compared to control group. In the blood of AP group compared to control group, 3-fold decreased GSH level was noted (Table 2).

**Figure 1:**
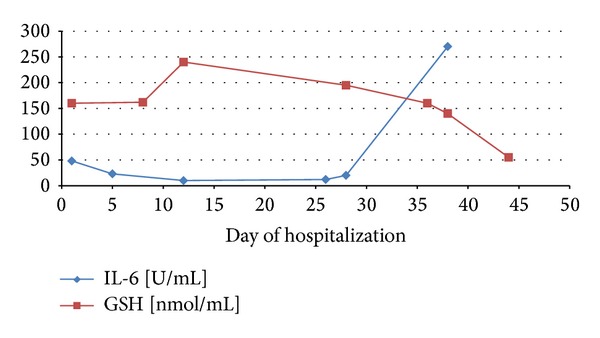
Dynamics of the changes in GSH level and IL-6 concentration in patient 6 with acute pancreatitis.

**Figure 2:**
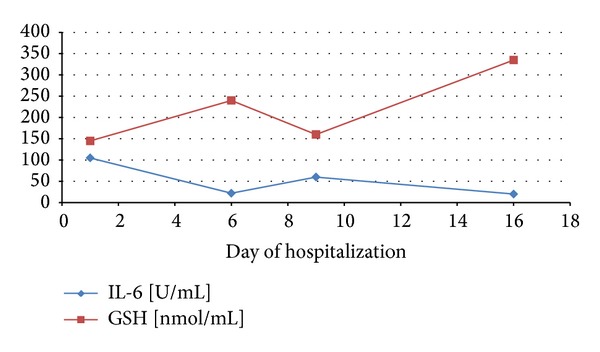
Dynamics of the changes in GSH level and IL-6 concentration in patient 2 with acute pancreatitis.

The dynamics of the changes in GSH level and IL-6 concentration in the case of selected patients were performed. It was shown that an increase in severity of pancreatitis caused a decrease in antioxidant status of organism in the serve AP (the patient died). An increase in IL-6 concentration which caused a decrease in GSH level was shown (Figure 1). In the other case, it was demonstrated that a lower concentration of IL-6 is accompanied by an increased GSH level (Figure 2). The dynamics of these parameters have shown that increased GSH level is beneficial to patient and it can lead to decreased IL-6 concentration.

### 3.3. Plasma MT and SH Groups Concentrations

A significant increase in MT concentration in the group of patients with AP was noted compared to control group. No differences in the concentration of SH groups between AP group and control group were shown (Table 2).

### 3.4. Plasma GPx Activity and Serum Cu/Zn SOD Activity

An increase in GPx activity in the plasma of patients with diagnosed AP compared to control group was observed. No statistically significant difference was detected in Cu/Zn SOD activity between the group of patients with AP and control group (Table 2).

The concentration of Cu and Zn participating in the maintenance of the stability of Cu/Zn SOD subunits was also measured. In the serum of control group, the values of Cu/Zn ratio ranged from 0.3 to 1.2. No difference between the group of patients with AP and control group was shown (Table 2).

### 3.5. Plasma TRAP Concentration and TBARS Level

No differences in the concentration of TRAP between examined groups were shown (Table 2). In this study, a statistically significant increase in the concentration of TBARS in AP group compared to control group was determined. The average concentration of this parameter in plasma of AP patients was over 2-fold higher as compared to the group of healthy volunteers (Table 2).

### 3.6. The Activity of Membrane Enzymes

AAP and GGT activities were measured. 11-fold increase in GGT activity in the group of patients with AP compared to control group was shown. However, no differences in AAP activity between examined groups were noted (Table 3). The analysis of AAP and GGT activities in depending on etiology has shown an increased AAP and GGT activity in idiopathic and gallstone AP compared to alcohol-related AP (Table 4).

### 3.7. The Activity of Lysosomal Enzymes

An increase in NAG activity in AP group compared to control group was demonstrated. In AP group, more than 3-fold increase in NAG activity was observed. However, no differences in *β*-GD activity between examined groups were shown (Table 3). In the AP group with gallstone and idiopathic etiology, an increased NAG activity compared to alcohol-related AP was shown (Table 4).

### 3.8. Correlations

In the group of patients with AP, the correlations between the parameters were observed (Table 5). The correlation between IL-6 concentration and Ranson criteria was also shown (Table 5). There were also the correlations between NAG activity and the concentration of IL-6 and activity of Cu/Zn SOD and GPx. The correlations between Cu/Zn SOD activity and IL-6 concentration and GPx activity were observed. Negative relations were noted between the GSH concentration and GGT activity and between GPx activity and TBARS concentration. There was a relation between GPx activity and the IL-6 concentration. The relation between activity of *β*-GD and NAG, *β*-GD and GGT, and AAP and GGT was demonstrated.

## 4. Discussion

The studies suggest that an essential mediator in the pathophysiology of acute pancreatitis is the inflammatory cytokines. A special role in initiating an inflammatory response in the course of acute pancreatitis was attributed to interleukin-6 (IL-6). IL-6 is a major inducer of acute phase protein synthesis in liver, and therefore an increase in this cytokine concentration in serum precedes (24 hours) the appearance of C-reactive protein [30]. Points of Ranson criteria commonly used to assess the patient's state only partly seem to confirm its usefulness as a predictor of disease course. Only four from seven patients, which died during hospitalisation as a severe case of acute pancreatitis (6.3 points in Ranson criteria), were classified. The disadvantage of Ranson criteria is also the fact that it is important parameter only in the first 48 hours after the occurrence of acute pancreatitis symptoms. The usefulness of Ranson criteria in later period of disease was not confirmed. Berney et al. [31] have confirmed that the method of determining the severity of pancreatitis on the base of serum IL-6 concentration (in the first three days of disease duration) is characterized by high specificity (67–95%), sensitivity (69–100%), and accuracy (80–84%). In addition, in our study, the positive correlation IL-6 with Ranson criteria was noted, which can confirm usefulness of IL-6 to assess acute pancreatitis severity.

In this study, was demonstrated over 55-fold increase in the level of IL-6 in the serum of patients with AP during the first 48 hours of hospitalization compared to the control group. In the study of Mayer et al. [1], it has been shown that high level of IL-6 is associated with increased mortality of patients with AP and it can be a marker for the development of systemic complications of pancreatitis. This study confirmed that an increase in severity of pancreatitis caused a decrease in GSH level in organism, which led to death of patient. A dramatic decrease in GSH concentration in this group of patients correlated with an increase in lipid peroxidation products (TBARS) (*P* < 0.01). A decrease in GSH level was similar to the results obtained by other researchers [32].

Significant changes in GSH level in the first phase of AP may be the result of massive attack of ROS and the participation of GSH in enzymatic elimination of oxidative stress products [32]. The findings obtained from the study confirm this assumption. A statistically significant increase in plasma GPx activity (participating mainly in GSH-dependent hydrogen peroxide elimination) was detected in patients with AP. A decrease in GSH level with a simultaneous increase in GPx activity has also found the confirmation in a statistically significant negative correlation (*P* < 0.001). This study suggests that a decrease in GSH level can be related to enhanced activity of GPx using GSH as substrate.

The studies on the people linking Cu/Zn SOD with pancreatitis turned out to be contradictory. It is possible that, in the course of acute pancreatitis, both GPx and Cu/Zn SOD were involved, but GPx/GSH system seems to play a role in the first defence line against ROS, whereas Cu/Zn SOD may play a role in chronic pancreatitis and its exacerbation. In the studies of Hausmann et al. a dramatic decrease in serum Cu/Zn SOD activity of patients with chronic pancreatitis was demonstrated [33]. A lowered enzyme activity in patients with severe AP than in those with mild pancreatitis was also shown [4]. It can explain no differences in Cu/Zn SOD activity in this study.

Zinc and copper ions participate in maintaining the proper stability of Cu/Zn SOD subunits and superoxidase radical anion neutralization. Kashimagi et al. have shown considerably lowered Zn concentration in pancreatic tissues in the course of pancreatitis [34]. Due to Zn participation in antioxidant processes, the authors suggested that Zn deficit might impair lipid metabolism and negatively influence the cell membrane protein synthesis. In present study, an increase in value of Cu/Zn ratio in the group of patients with AP compared to control group was not statistically significant. It can suggest that Zn deficiency in the group of patients with AP was not so considerable to cause the changes of Cu/Zn SOD activity, but it has influence on Cu/Zn imbalance. The Cu/Zn index can be increased and Cu may have a prooxidative effect. An increase in ROS production can be a cause of 2-fold increase in MT concentrations in AP group compared to control group. A significant increase in MT concentration in the plasma with AP (2.8 ng/mL) compared to control group (0.9 ng/mL) was shown [35]. Probably, MT is the protein, which plays an important role in the defence against oxidative stress. It may be considered as a sensitive marker, which protects before the changes in pro/antioxidant balance.

The pro/antioxidant imbalance in AP patients could lead to oxidative stress, which reflected an increase in TBARS concentration in the blood. An increase in lipid peroxidation products in the course of AP in its severe (6.2 *μ*mol/L) and mild form (4.0 *μ*mol/L) was also observed by Tsai et al. [36] and was later confirmed by the results of the further studies [37].

In our studies, the concentrations of SH groups and TRAP were measured, but the differences in AP group compared to control group were not statistically significant. It is contradictory in comparison to other studies, in which a decrease in SH groups concentration and an increased TRAP level in the group of patients with AP compared to healthy volunteers were shown [25]. It can suggest that the pro/antioxidant imbalance in acute pancreatitis can cause little changes in the level of TRAP, which indicate a small usefulness of TRAP determination. We observed an increase in levels of antioxidants, such as GPx or MT and the decrease in GSH concentration. These antioxidants seem to be important in the first line of defence against oxidative stress in acute pancreatitis.

The development of pancreatitis can cause the formation of numerous tissue damages and it can lead to enhanced release of enzymes from the cells and the extracellular space. In this study, membrane enzymes including AAP and GGT or lysosomal enzymes NAG and *β*-GD were measured.

In current study, an increase in GGT activity in patients with AP compared to control group can be caused by pancreatocytes damage as a result of pancreatitis or it can be an effect of increased demand for GSH in metabolism of organism, in which GGT is involved. Other results were contradictory. McKnight [38] suggested that the induction of GGT activity depends on bile stasis within the biliary tree. However, Hayakawa [39] has demonstrated that the increase of enzyme activity is result of alcohol abuse, which can cause an increase in enzyme release into serum (probably as a result of changes in cell membrane permeability). It was confirmed by other researchers which maintained that GGT determination can be a good marker differentiating alcohol-related AP from others AP [17]. In our study, the enhanced release of GGT into serum in gallstone and idiopathic AP compared to alcohol-related AP was demonstrated. It can indicate that AP is induced mainly by cholestasis and damage of duct epithelium. This process caused also the release of AAP in this group of patients, which can result in disorder of metabolic processes catalysed by AAP and GGT.

Inflammation can cause an increase in NAG level, what can be considered as a marker of lysosomal dysfunction and abnormal cellular integrity. In this study, more than 3-fold increase in NAG activity in serum of patients with AP was demonstrated. The analysis of AP patients in terms of etiology suggests that NAG was released into serum mainly as a result of pancreas damage during inflammatory process. It can indicate deep damages of pancreas, which led to total destruction of its structure.

The measurement of *β*-GD activity in examined groups did not show significant differences. In Wilson's studies [40], an increase in *β*-GD activity in the blood of rats with protein deficiency treated with ethanol was demonstrated. An increase in *β*-GD activity (but not statistically significant) in the group of patients with alcohol-related AP was also noted. This suggests that greater destruction of cells structure and lysosomal enzymes release in patients with gallstone and idiopathic etiology of AP compared to patients with alcohol related AP were observed.

In the patients with AP, the correlation between determinated parameters was shown. In the group of patients with AP, the correlation between IL-6 level and the activity of antioxidant enzymes: Cu/Zn SOD and GPx or between the IL-6 concentration and the activity of NAG was shown. The positive correlation of IL-6 concentration and NAG activity can confirm that inflammation increases tissue damage. The inflammatory state caused the increase in cytokine level and the running of antioxidant mechanism, wherein the correlations between antioxidant enzymes (GPx and Cu/Zn SOD) were negative. The relation between the activities of membrane enzymes (GGT and AAP) was noted, which can confirm the interaction of them, similar to the activities of lysosomal enzymes (NAG and *β*-GD). The membrane damages can lead to the enzymes release and an increase in the activity of AAP and GGT in serum. However, the destruction of cell organelles can lead to an increase in the activity of NAG and *β*-GD. The confirmation on the significant involving of GSH in the defence against oxidative stress was the increase in TBARS concentration and increased GPx activity, which correlated with a decrease in GSH level. With the development of the pancreatitis, the release of lysosomal enzymes, such as NAG, was increased. Its activity correlated with antioxidant enzymes activity (GPx and Cu/Zn SOD). An increased activity of GGT in the serum of patients with AP and a decreased level of GSH involving in the defence against free radicals in the negative correlation between these parameters were demonstrated.

## 5. Conclusions

**Figure 3:**
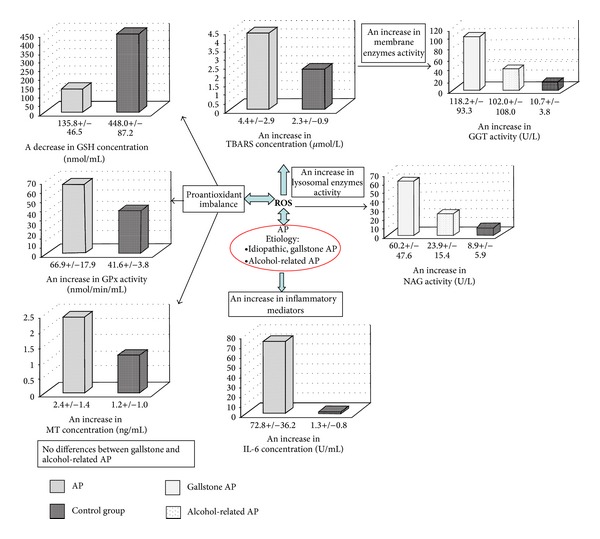
The graphical summary of the most important parameters determinated in the patients with acute pancreatitis compared to control group.

The main findings from the study were demonstrated in Figure 3. This study can be concluded as follows:

1. GSH, GPx, and MT seem to be the antioxidants the most involved in the defence against oxidative stress in acute pancreatitis.
2. A significant decrease in GSH level can be a result of increased GPx and GGT activities.
3. An increased TBARS concentration and the activities of membrane or lysosomal enzymes indicate deep tissue damage, more in gallstone and idiopathic AP than in alcohol-related AP.
4. The release of membrane and lysosomal enzymes in inflammatory process can contribute to metabolism dysfunction in nutrition and detoxification process, which can deteriorate the patient's condition.

## Figures and Tables

**Table 1:** IL-6 concentration and Ranson criteria as parameters describing the severity of acute pancreatitis.

Parameter	Control group *n* = 37	AP *n* = 32
IL-6 concentration [U/mL]	1.3	72.8∗
Standard deviation	0.8	36.2
Median	1.1	71.3
Range	0.3–2.5	21.5–143.4
Ranson criteria [points]	—	6.3
Standard deviation	—	1.7
Median	—	6.0
Range	—	4.0–9.0

*Statistically significant compared to control group.

**Table 2:** The parameters of pro/antioxidant balance in patients with acute pancreatitis compared to control group.

Parameter	Control group *n* = 37	AP *n* = 32
GSH concentration [nmol/mL]	448.0	135.8∗
Standard deviation	87.2	46.5
Median	432.9	140.5
Range	320.2–661.4	54.2–228.5
SH group concentration [umol/L]	294.3	219.0
Standard deviation	54.9	45.1
Median	309.0	227.0
Range	215.0–415.0	157.0–265.0
MT concentration [ng/mL]	1.2	2.4∗
Standard deviation	1.0	1.4
Median	0.8	2.1
Range	0.1–3.4	0.3–5.0
Cu/Zn ratio	0.7	0.9
Standard deviation	0.3	0.2
Median	0.8	0.9
Range	0.3–1.2	0.5–1.3
GPx activity [nmol/min/mL]	41.6	66.9∗
Standard deviation	3.8	17.9
Median	41.0	66.3
Range	36.3–49.7	33.1–86.8
Cu/Zn SOD activity [nmol/min/mL]	1.7	1.8
Standard deviation	0.3	0.5
Median	1.7	1.7
Range	0.9–2.2	0.8–2.6
TRAP concentration [umol/L]	1412.1	1268.7
Standard deviation	386.7	163.4
Median	1340.0	1233.0
Range	809.0–2097.0	1126.0–1447.0
TBARS concentration [umol/L]	2.3	4.4∗
Standard deviation	0.9	2.9
Median	2.3	3.7
Range	0.4–4.5	1.5–10.7

*Statistically significant compared to control group.

**Table 3:** The activity of membrane and lysosomal enzymes as a marker of tissue damage in patients with acute pancreatitis compared to control group.

Parameter	Control group *n* = 37	AP *n* = 32
GGT activity [U/L]	10.7	118.7∗
Standard deviation	3.8	79.8
Median	10.6	88.8
Range	6.8–22.7	41.8–295.8
AAP activity [U/L]	43.1	65.3
Standard deviation	10.9	46.9
Median	40.1	56.9
Range	27.5–66.6	29.2–228.1
NAG activity [U/L]	8.9	28.6∗
Standard deviation	5.9	22.4
Median	8.2	18.0
Range	1.5–24.3	8.1–91.4
*β*-GD activity [U/L]	1.3	1.4
Standard deviation	0.5	0.5
Median	1.3	1.4
Range	0.5–2.4	0.6–2.2

*Statistically significant compared to control group.

**Table 4:** Biochemical parameters in patients with acute pancreatitis related to gallstone and alcohol abuse (in the first 24 hours after admission).

Examined marker	Il-6 [U/mL]	NAG [U/L]	*β*-GD [U/L]	GGT [U/L]	AAP [U/L]
Gallstone and idiopathic acute pancreatitis					
Average/standard deviation	68.0 ± 41.8∗∗	60.2 ± 47.6^∗,∗∗^	1.3 ± 0.7	118.2 ± 93.3^∗,∗∗^	59.2 ± 11.2∗
Median	60.8	47.6	1.0	87.6	64.6
Range	20.2–143.4	17.8–126.3	0.5–2.2	46.2–295.8	44.3–68.4
The number of cases	12	12	12	12	12
Control group	1.3 ± 0.8	8.9 ± 5.9	1.3 ± 0.5	10.7 ± 3.8	43.1 ± 10.9

Alcohol-related acute pancreatitis					
Average/standard deviation	91.3 ± 79.0∗∗	23.9 ± 15.4∗∗	1.5 ± 0.4	102.0 ± 108.0∗∗	46.2 ± 16.2
Median	72.7	20.25	1.4	42.4	46.0
Range	20.2–143.4	4.2–48.2	0.6–2.2	17.4–328.8	20.9–72.2
The number of cases	20	20	20	20	20
Control group	21.5 ± 2.9	8.9 ± 5.9	1.3 ± 0.5	10.7 ± 3.8	43.1 ± 10.9

*Significant differences between gallstone and alcohol-related acute pancreatitis.

∗∗Significant differences between experimental and control group.

**Table 5:** Result of Spearman correlations for examined parameters in patients with AP.

Parameters	Ranson criteria	GSH	GPx	Cu/Zn SOD	AAP	NAG	*β*-GD
IL-6	0.71∗∗		0.60∗	0.55∗∗		0.73∗	
GPx		−0.70∗		−0.60∗			
TBARS		−0.76∗∗					
GGT		−0.75∗			0.65∗		−0.61∗∗
NAG			0.74∗	0.68∗			0.69∗

*Statistically significant for *P* < 0.001.

∗∗Statistically significant for *P* < 0.05.

## Abbreviations

AAP: Alanine aminopeptidase
AP: Acute pancreatitis
*β*-GD: *β*-Glucuronidase
Cu/Zn SOD: Superoxide dismutase
GGT: *γ*-Glutamyltransferase
GPx: Glutathione peroxidase
GSH: Reduced glutathione
IL-6: Interleukin 6
MT: Metallothionein
NAG: N-acetyl-*β*-D-glucosaminidase
ROS: Reactive oxygen species
TBARS: Thiobarbituric acid reactive substances
TRAP: Total peroxyl radical trapping potential.
